# Evaluating Explicit and Implicit Stigma of Mental Illness in Mental Health Professionals and Medical Students

**DOI:** 10.1007/s10597-014-9796-6

**Published:** 2014-12-23

**Authors:** Maciej Kopera, Hubert Suszek, Erin Bonar, Maciej Myszka, Bartłomiej Gmaj, Mark Ilgen, Marcin Wojnar

**Affiliations:** 1 Department of Psychiatry, Medical University of Warsaw, Nowowiejska 27, 00-665 Warsaw, Poland; 2 Faculty of Psychology, University of Warsaw, Ul. Stawki 5/7, 00-183 Warsaw, Poland; 3 Department of Psychiatry, Addiction Research Center, University of Michigan, Ann Arbor, MI USA

**Keywords:** Stigma, Mental illness, Implicit attitudes, Go/No-Go Association Task

## Abstract

The study investigated explicit and implicit attitudes towards people with mental illness among medical students (non-professionals) with no previous contact with mentally ill patients and psychiatrists and psychotherapists (professionals) who had at least 2 years of professional contact with mentally ill patients. Explicit attitudes where assessed by self-report. Implicit attitudes were measured with the Go/No-Go Association Task, a variant of the Implicit Association Test that does not require the use of a comparison category. Compared to non-professionals, mental health professionals reported significantly higher approach emotions than non-professionals towards people with mental illness, showed a lesser tendency to discriminate against them, and held less restrictive attitudes. Both groups reported negative implicit attitudes towards mentally ill. Results suggest that both non-professionals and professionals display ambivalent attitudes towards people with mental illness and that professional, long-term contact with people with mental illness does not necessarily modify negative implicit attitudes.

## Introduction

Current research indicates that mental illness is often related to social stigmatization, discrimination and prejudice and that people with mental illness are often perceived as dangerous, unpredictable and aggressive (Rüsch et al. 2005; Hinshaw and Stier 2008). Despite some research reporting positive evaluations and attitudes toward people with mental illness (e.g., Granello and Wheaton 2001; Link and Phelan 1999) and increasing public knowledge about mental illness, a recent meta-analysis of changes in public attitudes during the last 20 years, revealed that attitudes towards people with mental illness have not significantly improved (Schomerus et al. 2012). The present study was conducted in Poland where studies performed by Public Opinion Research Center (Wciórka and Wciórka 2005, 2008) have found that respondents help positive attitudes toward people with mental illness; however, they also believed that social attitudes are generally negative.

Stigmatization is considered an influential factor contributing to high dropout rates in the treatment of psychiatric population (Rüsch et al. 2005). People with mental illness who internalize negative attitudes toward mental illness may subsequently avoid seeking treatment (Sirey et al. 2001). Apart from the consequences of self-stigmatization (Corrigan et al. 2006), individuals with mental illness may be reluctant to seek professional due to anticipation of encountering negative bias within the psychiatric healthcare system (Link and Phelan 2001). Thus, it is important to understand the phenomenon of stigma and negative attitudes toward mental illness among the mental health professionals who are responsible for providing care to individuals with mental illness.

Education and contact with persons with mental illness are associated with reduced stigma (Rüsch et al. 2005). However, results of studies evaluating differences in explicit attitudes towards people with mental illness between mental health professionals and the general population are inconsistent. For example, Kingdon et al. (2004) found that psychiatrists’ attitudes toward people with mental illness were more positive compared to members of the general population (see also Vibha et al. 2008). On the contrary, Nordt et al. (2006) reported that psychiatrists had more negative attitudes towards patients with schizophrenia and major depression than a sample from the general population (see also Aydin et al. 2003; Hansson et al. 2013).

Such conflicting results may be, at least in part, due to the use of self-report assessment measures which introduce the potential for social desirability bias. Importantly, self-report measures limit our assessment to that part of the attitude structure of which we are aware and do not necessarily assess implicit components of attitudes, which reside outside our conscious control (for rev. Greenwald and Nosek 2001). As defined by Greenwald and Banaji (1995) implicit attitudes are “introspectively unidentified (or inaccurately identified) traces of past experience that mediate favorable or unfavorable feelings toward an attitude object” (p. 6). Measures of implicit cognition provide the opportunity to reduce deliberate judgment and decrease the probability that study participants can hide undesired responses due to social desirability. As evidenced by social science research, in some cases, explicit and implicit biases are unrelated and in other cases they are strongly correlated (Nosek et al. 2002). Consequently implicit and explicit biases seem to be different predictors of measured behavioral manifestations.

Although there has been a great deal of research concerning explicit attitudes toward people with mental illness, evaluations of implicit cognitions are limited, especially among mental health professionals. One study found that mental health professionals demonstrated more *positive* implicit and explicit evaluations of people with mental illness than did the groups with little or no mental health training (general public and undergraduates), and those with alternate health care experience (other health/social services professions) (Peris et al. 2008). Studies among other samples regarding attitudes toward individuals with mental illness have reported negative attitudes. For example, among college students, Teachman et al. (2006) have established evidence for both implicit and explicit negative biases against persons with mental illness. Similarly, O’Driscoll et al. (2012) reported that children and adolescents both implicitly and explicitly stigmatize peers with attention-deficit/hyperactivity disorder and depression. In the large group of undergraduate students Monteith and Pettit (2011) confirmed, that implicit responses to depression were more negative when compared to physical illness as a contrast condition. Data also suggest that negative implicit attitudes toward mental illness are also manifested by people with mental illness (Teachman et al. 2006) and are related to lower quality of life in this group (Rüsch et al. 2010).

Although these prior studies provide useful information regarding implicit attitudes toward people with mental illness, they are also limited by their methodology for assessing implicit attitudes. Most often, studies of this nature use a modification of the Implicit Association Test (IAT). The IAT method employs a contrasting category (e.g., physical illness in Teachman et al. 2006 or Monteith and Pettit 2011 and welfare recipients in Peris et al. 2008); thus, measurement of implicit attitudes toward one group is biased by the selection of contrasting category. Context independent measures, such as the Go/No-Go Association Task (GNAT; Nosek and Banaji 2001), provide an alternative means of assessing this implicit stigma without the inherent limitation of a contrast category.

Considering prior research and the limitations of the IAT method, the purpose of the current study was to investigate explicit and implicit attitudes towards people with mental illness among mental health professionals. We also assessed how professional contact with patients with mental illness was related to both explicit and implicit attitudes. Specifically, we developed the present study to compare medical students (non-professionals) who never had professional or long-term contact with mentally ill patients with psychiatrists and psychotherapists (professionals) who had at least 2 years of professional contact with patients with mental illness.

## Methods

### Participants and Procedure

The study was performed in the Department of Psychiatry at the Medical University of Warsaw and the research protocol was approved by the Bioethics Committee of the Faculty of Psychology, University of Warsaw. All measures and procedures were administered in Polish. Participants included two groups: professionals and non-professionals, all of whom provided written informed consent. The professionals group consisted of 29 psychiatrists and psychotherapists recruited among the personnel of the Department of Psychiatry, including 15 females and 14 males. All professionals had at least 2 years of clinical experience working with mentally ill patients. The non-professional group consisted of 28 first-year medical students, including 10 females and 18 males. The students were tested during the integration camp preceding the beginning of the first year. Based on a preliminary screening, only the students who reported no previous personal contact with mentally ill people (member of family, friends, neighbors) were eligible for the study. The participation in the study was voluntary and participants were not compensated. All participants completed the study individually, in a private room. Participants first completed a series of the set of questionnaires (described below), followed by the GNAT task using an IBM computer.

### Measures

#### Explicit Attitudes

Participants completed two measures of explicit attitudes toward people with mental illness. The first measure (Emotion Scale) was constructed by the authors for the present study in order to assess emotions that may be experienced during contact with a person with mental illness. Participants were instructed to imagine contact with a person with mental illness (without exact specification of the type of illness) and to then rate the intensity of 10 emotions they could experience during this encounter. Nine-point Likert scales (ranging from 1 = weak to 9 = strong) were used to assess the intensity of ten emotions: compassion, interest, sadness, acceptance, anger, dislike, anxiety, aversion, distrust and indifference. The selection was driven by the assumption that these emotions are frequently experienced during the encounter with mentally ill individuals.

Participants also completed the 51-item Opinions about Mental Illness Scale (OMI; Cohen and Struening 1962), which assesses several dimensions of explicit attitudes toward people with mental illness. This measure included subscale scores for the following domains: authoritarianism (tendency to perceive those with mental illness as a class of people inferior to normal individuals and requiring coercive handling), social restrictiveness (desire to protect the society by restricting mental patients both during and after hospitalization), benevolence (encouraging or nurturing attitude), mental hygiene ideology (need to encourage equal social participation and incorporation of people with mental illness into every aspect of community life), and interpersonal etiology (belief that mental illness arises from negative interpersonal experiences, such as lack of parental love and attention during childhood). Participants had to rate each statement on 6-point Likert scale ranging from strongly agree to strongly disagree. Total scores for each subscale ranging from: 1 to 46 for mental hygiene, 1–51 for social restrictiveness, 1–66 for benevolence, 1–56 for authoritarianism, and 1–36 for interpersonal etiology. The OMI was translated into Polish by the authors and back-translated by a native English speaker. Differences were discussed until a consensus translation was obtained. Reliability coefficients for each subscale reported by Cohen and Struening (1962) ranged from alpha = 0.38 (mental hygiene) to 0.89 (authoritarianism). In the present sample estimates of Cronbach’s alpha were similar. Specifically, alphas were 0.43 (mental hygiene), 0.7 (social restrictiveness), 0.4 (benevolence), 0.6 (authoritarianism), 0.70 (interpersonal etiology).

#### Implicit Attitudes

The GNAT (Nosek and Banaji 2001) was used to measure automatic associations between concept (e.g., mental illness) represented by the target category and attribute (e.g., evaluation) categories. In our study, the GNAT consisted of two blocks. In one block, the target category (mentally ill) was paired with an attribute (e.g., pleasant) and in the other block, the target category was paired with the opposing attribute (e.g., unpleasant). In our version of the test, the distracter (noise) items reflected various professions (e.g., journalist) and the opposite attribute (e.g., when ‘unpleasant’ was signal, ‘pleasant’ was noise). During GNAT administration, labels of the target categories appeared and remained on the screen in the upper left and right quadrants, reminding the target category and target attribute for a particular block. Participants were instructed to press the space bar as quickly as possible (Go) for items belonging to either of the categories which were displayed on the screen in each particular block. If the item did not belong to either category they were instructed to withhold the response (No-Go). For the trials where the participant correctly responded to an item treated as signal (hits) or did not respond noise items (correct rejections) a green “O” appeared on the screen during the inter-item interval to provide feedback about performance accuracy (these trials were scored as correct responses). On trials where noise items were incorrectly categorized as signal (false alarms) or signal items were not categorized (misses) a red “X” appeared below the stimulus item (these trials were scored as errors). The target category (mentally ill) contained words that describe mentally ill persons, taken from a Polish population based national survey performed by The Public Opinion Research Center (CBOS) (Wciórka and Wciórka 2005). From the set of attributes describing mentally ill patients selected by the respondents, we decided to include only words which were categorized as having neutral valence (e.g., schizophrenic, depressive, neurotic).

### Data Analysis

The Emotion Scale was subjected to the factor analysis. All eigenvalues were higher than 1. Principal components analysis with oblimin rotation and the inspection of the scree plot revealed two factors. The rotated solution revealed simple structure with all variables loading only on one factor. The first one accounted for 33 % of the total variance, with eigenvalue = 3.9 and consisted of the items including anger, dislike, anxiety, aversion, distrust, indifference. Based on the content of the items loading above 0.3 on this factor we labeled the subscale “Withdrawal Emotions.” Cronbach’s alpha for these items was 0.77. The second factor labeled, “Approach Emotions,” accounted for 16 % of variance, with eigenvalue = 1.9 and encompassed compassion, interest, sadness, acceptance. Cronbach’s alpha for these items was 0.63. Scores on items within each component were averaged together to create a mean subscale score. The between-group differences (i.e., professional vs. non-professional) in explicit attitudes were assessed with Student’s *t* test for independent samples.

The GNAT data were scored according to the algorithm recommended by Nosek and Banaji (2001). We applied the measure of sensitivity indexed by d-prime (d′), which represents the ability to discriminate targets (signal) from distracters (noise). The proportion of hits and false alarms was converted into z-scores, and the difference between *z*-score values for hits and false alarms was indexed as d′. It is assumed that greater sensitivity indicates stronger association between the target category (e.g., mentally ill) and the attribute (e.g., pleasant vs. unpleasant). The Student’s *t* test for dependent samples was used for comparing the strength of positive versus negative associations within each group.

The criterion for statistical significance in all tests was *p* < 0.05. The data were analyzed using SPSS^®^ 18.0 for Windows.

## Results

Analyses examining whether professional and non-professional groups differed in terms of age and gender revealed that professionals were significantly older than non-professionals in age (*M* = 29.52, *SD* = 3.38 vs. *M* = 19.21, *SD* = 0.86; *t*(29) = −15.4; *p* < 0.001). However, there were no significant between group differences in gender ratio (χ^2^ = 1.48; *p* = 0.22). In both study groups, age was not significantly correlated with explicit and implicit attitudes measures.

Both non-professionals and professionals declared significantly higher intensity of approach than withdrawal emotions toward mentally ill individuals [*M*_approach_ = 5.77, *SD* = 1.64 vs. *M*_withdrawal_ = 3.01, *SD* = 1.35; *t*(28) = 6.52; *p* < 0.001 for non-professionals; *M*_approach_ = 6.76, *SD* = 1.16 vs. *M*_withdrawal_ = 2.59, *SD* = 1.08; *t*(30) = 13.83; *p* < 0.001 for professionals]. Professionals reported significantly higher intensity of approach emotions than non-professionals [*M* = 6.76 vs. *M* = 5.77; *t*(47) = −2.75; *p* < 0.02]. The two groups did not differ significantly in the intensity of withdrawal emotions [*t*(58) = 1.38; *p* = 0.07].

Analysis of the results from the OMI Scale revealed that professionals obtained significantly lower scores than non-professionals on the authoritarianism [*M* = 17.04, *SD* = 4.23 vs. *M* = 21.27, *SD* = 5.44; *t*(58) = 3.59; *p* = 0.001] and social restrictiveness [*M* = 15.83, *SD* = 4.54 vs. *M* = 22.15, *SD* = 5.79; *t*(58) = 4.08; *p* < 0.001] subscales. There were no between-group differences on the OMI scales measuring benevolence [*t*(46) = −1.46; *p* = 0.15], mental hygiene ideology [*t*(57) = −0.51; *p* = 0.56] and interpersonal etiology [*t*(58) = 1.55; *p* = 0.13].

With regard to implicit attitudes as measured by the GNAT, both groups of participants showed greater sensitivity to *mentally ill* + *unpleasant* than to *mentally ill* + *pleasant,* suggesting a negative implicit attitude toward people with mental illness. This effect was observed for both non-professionals [*M*_unpleasant_ = 2.4, *SD* = 0.89 vs. *M*_pleasant_ = 1.73, *SD* = 0.77; *t*(29) = −7.05; *p* < 0.001; Cohen’s *d* = 0.8] and professionals [*M* = 2.21_unpleasant_, *SD* = 0.78 vs. *M*_pleasant_ = 1.89, *SD* = 0.71; *t*(26) = −3.28; *p* < 0.003; Cohen’s *d* = 0.4]. There were no between-group differences among professionals and non-professionals in sensitivity to *mentally ill* + *unpleasant* [*t*(55) = 0.84; *p* = 0.4] and *mentally ill* + *pleasant* [*t*(56) = −0.84; *p* = 0.4].

## Discussion

The current study investigated implicit and explicit attitudes toward individuals with mental illness in Poland among professionals with mental health training (psychiatrists or psychotherapists with at least 2 years of professional experience in clinical work) and first year medical students reporting no personal contact with mental illness (i.e., non-professionals). We investigated whether long-term professional contact with patients with mental illness was associated with differences explicit and implicit attitudes compared to having no professional or personal contact with individuals with mental illness. Results indicated that both groups self-reported positive explicit attitudes towards individuals with mental illness. There are several possible interpretations of this finding.

First, it is plausible that informational and educational campaigns and programs to reduce stigma related to mental illness introduced in Poland in the recent years have been successful (e.g., Open the Doors program of the World Psychiatric Association since 2000). Thus, these favorable attitudes may be a result of a societal shift of self-reported evaluations in a positive direction, however the absence of an experimental design precludes testing this hypothesis.

Alternatively, these results may reveal a need for social approval by members of the study groups. According to social norms, individuals with mental illness are supposed to be the object of tolerance and acceptance, and it is not appropriate and socially acceptable to express prejudice overtly (e.g., Teachman et al. 2006), and this belief may be even stronger among those in the helping professions. For example, when looking at the between-group differences, professionals declared more positive explicit evaluations, expressed in higher approach emotions, less authoritarianism and less social restrictiveness than non-professionals. Following the social desirability effect, professionals’ responses may be even more distorted than students’ responses in terms of what they perceive to be professionally desirable. Also, these results can be interpreted through the lens of the contact hypothesis (Pettigrew and Tropp 2005) which suggests that interaction with members of stigmatized groups works to reduce stigmatizing attitudes and behaviors. There is some evidence, that personal contact with people with mental illness may reduce stigma (Ogedengbe 1993; Penn et al. 1994; Peris et al. 2008), however, opposite results have been also reported (Murphy et al. 1993; Weller and Grunes 1988). Finally, it is also possible that that the more positive evaluations reported by professionals may reflect generally more favorable views that these individuals had even before entering the mental health field.

In contrast, there were no between group differences on implicit attitudes towards the category “mentally ill.” Analysis of the GNAT results revealed that participants were more likely to associate mental illness with negative than positive attributes. The effect sizes were high for non-professionals (Cohen’s d = 0.8), and moderate for professionals (Cohen’s d = 0.4). Our results are in contrast with Peris et al. (2008) findings which showed positive implicit attitudes toward mental illness which were significantly influenced by the level of professional mental health training (i.e., more training was related to less stigma). It is plausible, that this discrepancy between the studies is due to the different measurement strategies to appraise implicit mental illness stigma and results from the selection of the comparison category. Using *welfare recipients* as comparison category in the IAT, Peris and colleagues introduced another stigmatized group as the reference for the people with mental illness. Despite the well-documented utility of the IAT (Greenwald et al. 2009) as a measure of implicit attitudes it is not possible to interpret its results irrespective of the contrasting category. In this case the evaluations of persons with mental illness depended on the evaluations of *welfare recipients.* Alternatively, the GNAT as utilized as a measure of automatically activated evaluation in our study, does not depend on the selection of the comparison category and is therefore free from this particular kind of bias. The present results are consistent with those negative implicit attitudes towards individuals with mental illness measured with the IAT among non-professionals as reported in Teachman et al. (2006), as well as towards depression in Monteith and Pettit (2011). However, it is also important to note that in both studies a comparison category (*physical illness)* was utilized.

Our study results also indicate that attitudes toward people with mental illness are ambivalent both among students and professionals. Observed dissociations between explicit and implicit attitudes have been commonly reported in the studies on attitudes toward other stigmatized groups, such as members of racial, ethnic, and sexual minorities (Rudman et al. 1999; Devos and Banaji 2005; Steffens 2008). Likewise, individuals with mental illness might be the object of the same attitudinal dissociation. This may vary based on level of contact and professional training. However despite clearly positive self-reported evaluations among both professionals and non-professionals, both groups’ implicit attitudes indicated a negative bias. When intentional control over behavior was eliminated, non-professionals tended to associate mentally ill with negative evaluations. Because, lay concepts of mental disorder are molded by episodic and tendentious information presented by media or culturally inherited myths and stereotypes (Wahl 1995), it is not surprising that these non-professionals who lacked personal contact with mental illness had implicit biases.

Yet more intriguing is similar dissociation observed between explicit and implicit attitudes in professionals. Our results suggest that despite the therapeutic training, professional knowledge, and everyday contact, which are the factors that presumably should counteract biased views of mental illness, implicit evaluations were negative. This outcome may indicate the persistence of implicit attitudes. Contrary to our study results, prior research with mental health care professionals reported more positive implicit evaluations compared to groups with little or no mental health training, and those with other health care experience (Peris et al. 2008). Alternatively, Teachman and Brownwell (2001) reported strong implicit anti-fat bias among health professionals who specialize and work with obese patients. Automatic evaluations of overweight persons as bad and lazy were also prevalent in this group. However, it is important to note that both our results and those of prior studies evaluating implicit and explicit bias may be influenced according to possible moderators such as interpersonal factors (e.g., self-presentation) or intrapersonal factors (e.g., amount of personal experience with a particular domain; Nosek 2007), or other factors such as research measurement, design, and sampling procedure. Further, negative implicit attitudes among professionals, may reflect the unconscious emotions related to clinical work. Among other medical professions, mental health professions are considered to be lower in status (Hinshaw and Stier 2008). Working with individuals with mental illness is often described as difficult and prone to burnout. Moreover, the salaries of mental health workers are on the low end of ranges for the general health staff (Hinshaw and Stier 2008). Thus, it is plausible that these factors influence professionals’ attitudes toward their patients.

Despite these implications, there are several limitations of the present study. The study sample was relatively small and thus caution must be exercised when generalizing the findings. The mental health professionals were on average 10 years older than the medical students. Moreover, the selected category (“mentally ill”), may be far more heterogeneous for mental health professionals as compared to the general population. For example, psychiatrists and psychotherapists work with patients suffering from various mental health problems; thus, there could be variations in implicit and explicit attitudes depending on presentation and diagnosis (e.g., depression, anxiety disorders, personality disorders, or schizophrenia). Therefore, when assessing bias in this specific population more information might be obtained by evaluating attitudes within a diagnostic category. Furthermore, it is plausible that our use of the category “mentally ill” is a subcategory within the wider (and more general) “ill” category. Assuming that neutral (without emotional valence) representations of the given category does not influence GNAT results (Nosek and Banaji 2001), participants may automatically react to the “ill” as target category and it seems unlikely that people may associate “ill” with “good” attributes. Furthermore, even if the category “mentally ill” is associated with more positive attitudes, automatic behavioral reactions might be driven more by a general tendency to react to the concept of “illness” which is clearly related to suffering. Furthermore, it is important to note that in our study, we could not experimentally manipulate personal and/or professional contact with individuals with mental illness, therefore causality cannot be determined. Further, results may have been biased by demand characteristics. Finally, the study was performed in the University Clinic and results may not be generalizable to professionals and non-professionals from other settings.

The present results suggest several practical implications. Given the discrepancy found between explicit and implicit attitudes as well as previous reports (e.g., Teachman et al. 2006), explicit measures might be strongly influenced by social desirability and, therefore, may not adequately assess significant dimensions of the attitude construct. Relying only on the self-report measures provides information about how people believe they should feel, but may still be in contrast to their behavior. As the data from the domain of racial prejudice indicate, implicit bias may predict prejudiced behaviors more effectively than self-report (e.g., Dovidio et al. 2002, 2007). Following the Peris et al. (2008) study results, implicit and explicit biases toward mental illness may predict different clinical decision-making. Corrigan et al. (2002), found that fear of dangerousness negatively predicted helping behavior toward individuals with mental illness. In light of the negative automatic bias revealed in professionals in our study it would be reasonable to expand knowledge concerning stigmatization in mental health care professionals during their training and look for the new strategies to modify it. This knowledge may reduce bias and prevent discrimination. Educational programs, anti-stigma campaigns and personal contact with mentally ill individuals are still considered to be important factors for reducing stigma; however, it is possible that all these actions influence only the explicit level of attitudes and do not impact unintentional processes grounded in the implicit bias. Consequently, community-based stigma reduction programs and campaigns may not be fully effective and therefore future research must evaluate methods for reducing implicit biases as well.
